# Age‐ and Sex‐Dependent Dynamics in Pituitary Thyrotroph and Thyroid Hormones in Dromedary Camels (*Camelus dromedarius*): A Histochemical Approach

**DOI:** 10.1002/vms3.71007

**Published:** 2026-05-29

**Authors:** Shaukat Ali Shaukat Jaspal, Muhammad Mubashar Shaukat, Robina Shaukat, Tahmina Shaukat, Rifat Ullah Khan, Shabana Naz, Antonella Perillo, Naseer Khan Momand, Ibrahim A. Alhidary

**Affiliations:** ^1^ Institute of Zoology University of the Punjab Lahore Pakistan; ^2^ Department of Zoology, Wildlife and Fisheries University of Agriculture Faisalabad Pakistan; ^3^ Physiology Lab, College of Veterinary Sciences, Faculty of Animal Husbandry & Veterinary Sciences The University of Agriculture Peshawar Pakistan; ^4^ Department of Zoology Government College University Faisalabad Pakistan; ^5^ Department of Veterianry Medical Sciences Alma Mater, Studiorum, Univeristy of Bologna Bologna Italy; ^6^ Department of Animal Production, College of Food and Agriculture Science King Saud University Riyadh Saudi Arabia

**Keywords:** age, dromedary camel, lactation, thyroid hormones, thyrotrophs

## Abstract

**Background:**

The hypothalamic–pituitary–thyroid axis plays a crucial role in regulating metabolism and reproduction, yet its age‐ and sex‐related dynamics in dromedary camels remain poorly understood.

**Objective:**

This study aimed to investigate age‐ and sex‐dependent variations in pituitary thyrotroph morphometry and thyroid hormone profiles in dromedary camels.

**Methods:**

A total of 90 clinically healthy Brela breed camels of both sexes (aged 2 to ≥11 years) were grouped based on age and physiological status (lactating and non‐lactating). Pituitary tissues (*n* = 60) were subjected to immunohistochemical analysis for thyroid stimulating hormone (TSH)‐producing thyrotrophs. Morphometric parameters, including cell count, size, and nuclear dimensions, were measured. Plasma concentrations of TSH, triiodothyronine (T_3_), and thyroxine (T_4_) were determined using ELISA.

**Results:**

Thyrotroph cell counts did not differ significantly among groups. However, older camels (≥11 years) showed significantly increased cell and nuclear dimensions (*p* < 0.05). Plasma TSH levels decreased with age, with peak levels observed in females aged 5–10 years. T_3_ concentrations varied significantly with age and sex, with higher levels in younger males. T_4_ levels declined with age but were not significantly influenced by sex (*p* > 0.05).

**Conclusion:**

Thyrotroph morphology and thyroid hormone dynamics in dromedary camels are significantly influenced by age and sex, indicating adaptive modulation of the hypothalamic–pituitary–thyroid axis in response to metabolic and reproductive demands.

## Graphical abstract

This study demonstrates age‐ and sex‐dependent regulation of the hypothalamic pituitary thyroid axis in dromedary camels, showing enlarged thyrotroph morphology in older animals and distinct hormonal patterns, including declining T_4_ with age‐ and sex‐related variation in T_3_ and TSH levels.

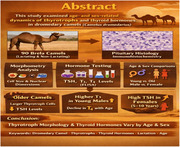

## 1 Introduction

Recent advancements in animal research have highlighted the critical role of animal models in both biomedical and veterinary sciences, particularly in understanding physiological mechanisms and supporting surgical and implant‐based innovations (Choudhary 2025). Animal models not only facilitate translational research but also enhance anatomical and histophysiological investigations across species, while also serving as effective tools in veterinary education and anatomical training (Choudhary and Sarkar 2025). Furthermore, emerging technologies such as artificial intelligence (AI) are increasingly being integrated into animal anatomy and diagnostic sciences, offering novel opportunities for data analysis, imaging interpretation, and predictive modelling (Choudhary 2026). Incorporating these modern approaches strengthens the scientific framework for investigating endocrine dynamics, such as pituitary–thyroid interactions, in species like the dromedary camel.

The dromedary camel (*Camelus dromedarius*) plays a crucial role in sustaining livelihoods in arid and semi‐arid regions, where it provides milk, meat, transport, and economic stability (Babar and Ashraf 2023; Tharwat et al. 2025). Unlike other domestic livestock, camels are uniquely adapted to withstand environmental challenges such as heat stress, water scarcity, and feed limitation. These adaptive traits are underpinned by physiological, behavioural, and endocrine regulatory mechanisms, enabling survival and productivity under desert conditions (Hassan et al. 2025; Mohammed et al. 2025).

Among the endocrine systems, the hypothalamic–pituitary–thyroid (HPT) axis is central to metabolic regulation and thermoregulation in mammals. Thyrotrophs of the adenohypophysis secrete thyroid stimulating hormone (TSH), which drives thyroid hormone synthesis and modulates energy metabolism, reproduction, and lactation (Al‐Suhaimi and Khan 2022). Morphological changes in thyrotrophs often reflect functional alterations in hormone production during different physiological states such as growth, aging, and lactation (Duntas 2021).

Lactation, a physiologically demanding stage, requires major endocrine adjustments to support milk synthesis and energy partitioning. Studies in dairy animals demonstrate structural and functional modifications in pituitary cells during lactation (Gul et al. 2024). However, such adaptations remain largely unexplored in camels, despite the nutritional and therapeutic value of their milk. Similarly, advancing age is known to alter pituitary cell morphology and endocrine capacity, potentially reducing reproductive efficiency and milk yield in camels (Mostafa et al. 2022).

Morphometric evaluation of pituitary thyrotrophs provides an indirect indicator of their functional state, with increased cell size, nuclear volume, and cytoplasmic area often associated with heightened activity. Immunocytochemical techniques allow precise characterisation of these parameters. While such approaches have been applied in ruminants and poultry (Gnanadevi et al. 2025; Goralsky et al. 2021; Jaspal et al. 2019; Khan et al. 2013), equivalent studies in camels are lacking. Previous camel research has focused mainly on circulating thyroid hormones or thyroid gland histology (Ahmed et al. 2016; Rejeb et al. 2011), leaving the pituitary contribution to thyroid regulation poorly defined. No study has compared adenohypophyseal thyrotroph morphology in lactating and non‐lactating she‐camels across different age groups. Therefore, this study was undertaken to evaluate the morphometric characteristics of adenohypophyseal thyrotrophs in lactating and non‐lactating *Camelus dromedarius* across three age groups (2–4, 5–10, and ≥11 years). This approach provides new insights into the endocrine adaptations of the dromedary camel to physiological state and age, contributing to a better understanding of reproductive and metabolic regulation in this desert‐adapted species.

## 2 Materials and Methods

### 2.1 Experimental Animals

A total of 90 apparently healthy dromedary camels (*Camelus dromedarius*) were examined and sampled during the peak breeding season. All animals were sourced from a single commercial camel farm that supplies the municipal abattoirs, ensuring a uniform feeding and management background. They were categorised into three age groups: 2–4 years, 5–10 years, and ≥ 11 years, with 15 males and 15 females per group. Age was determined by the management records from the farm staff for each animal. The rationale for this age‐based classification is that 2–4 years generally encompasses the peri‐pubertal to early‐reproductive stage, 5–10 years corresponds to prime reproductive maturity, and ≥ 11 years represents late maturity or senescence. Previous work indicates that female dromedaries typically attain puberty between 3 and 4 years, while males reach full sexual maturity at approximately 4–6 years but can exhibit rutting behaviour earlier under favourable management.

Because all animals originated from the same farm with a consistent feeding regimen (grazing supplemented with concentrate and mineral mix), we minimised potential confounding from nutritional variation. To further reduce misclassification related to reproductive status, we incorporated direct reproductive assessments (e.g., ovarian and testicular examination at slaughter) in addition to age. This combined approach supports the validity of our three‐tier grouping while allowing meaningful comparison with earlier endocrine and morphometric studies that used similar age cut‐offs.

### 2.2 Assessment of Reproductive Status

To minimise sampling error related to reproductive physiology, each animal underwent a brief clinical and reproductive tract evaluation immediately after slaughter. In females, the ovaries and uterus were inspected to confirm the presence of follicles, corpora lutea, or other evidence of cyclic activity. Males were examined for testicular size, consistency, and the presence of characteristic rutting signs (e.g., poll gland enlargement, neck muscle hypertrophy). Only animals with no gross pathological lesions and with normal reproductive organs were included. This combined clinical examination and age verification ensured that the categorised animals were indeed reproductively representative of their assigned groups, addressing concerns about potential misclassification and strengthening the biological basis for age‐group comparisons.

### 2.3 Sample Collection

Pituitary glands were collected from 60 clinically normal camels using the extirpation technique described by Jaspal et al. (2019). To prevent hormone diffusion and loss, samples were harvested within 4 h post‐slaughter.

### 2.4 Tissue Preparation and Immunohistochemistry

**FIGURE 1:**
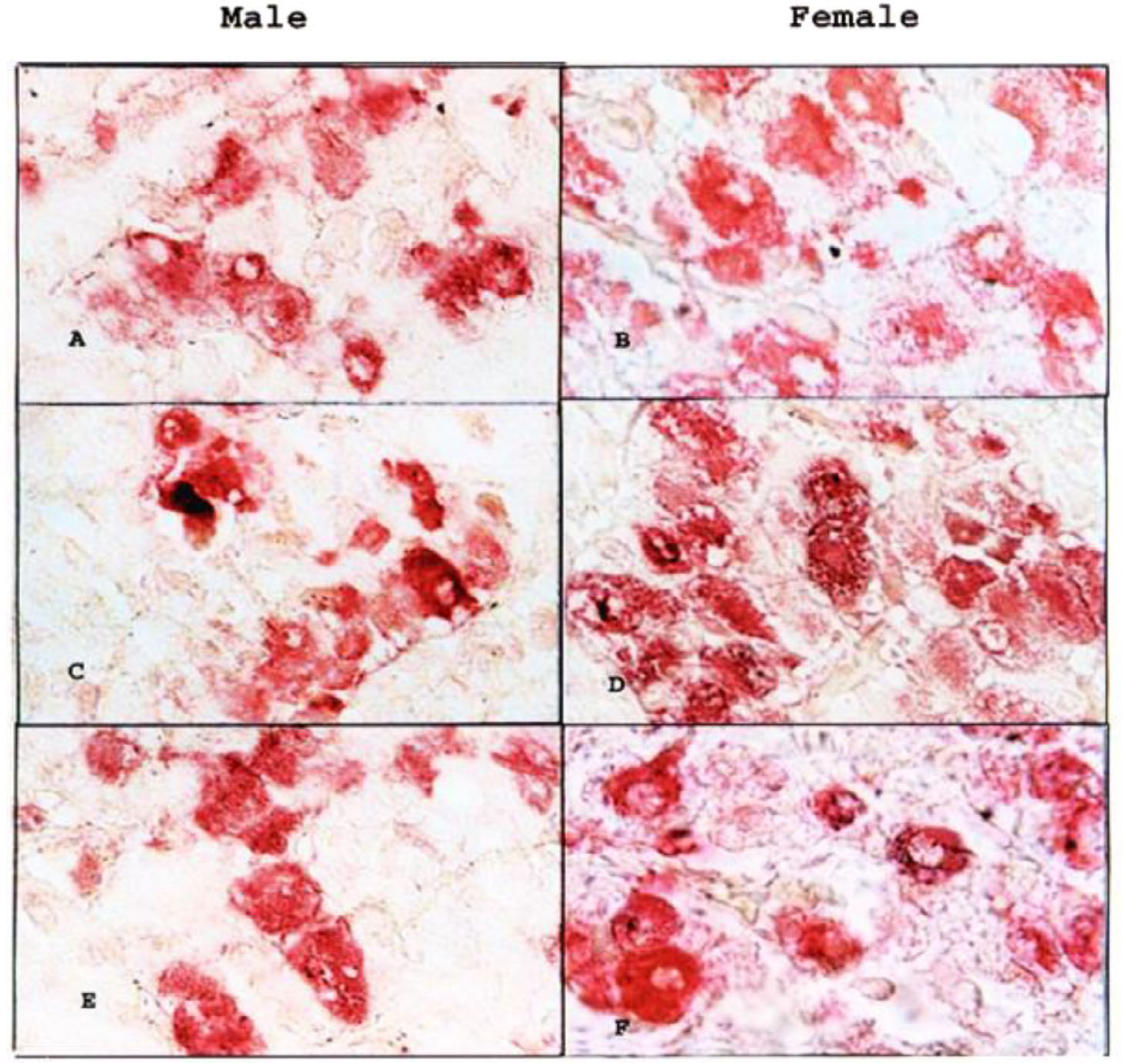
Thyroid stimulating hormone (TSH) immunoreactive cells in the adenohypophysis of camel (*Camelus dromedarius*). Streptavidin–alkaline phosphatase immunoreaction: X5000. **(A)** 2–4 years male: immunoreactive cells small spherical rather than elongated with small nuclei. **(B)** 2–4 years female: immunoreactive cells elongated or polygonal with large nuclei. **(C)** 5–10 years male: immunoreactive cells spherical with spherical nuclei. **(D)** 5–10 years female: immunoreactive cells polygonal with irregular nuclei. **(E)** 11–onward years male: Immunoreactive cells polygonal with small irregular nuclei. **(F)** 11–onward years female: immunoreactive cells large spherical with spherical nuclei in the centre.

Pituitary glands were fixed in Bouin's solution, dehydrated using standard protocols, and embedded in freshly melted paraffin wax. Midsagittal sections (4 µm thick) were prepared using a rotary microtome (Leica RM‐2235) and mounted on Poly‐L‐Lysine–coated slides (Sigma, St. Louis, MO, USA). Slides were stored in dust‐proof boxes for 7 days prior to analysis. For immunohistochemistry, slides were immersed in Lugol's iodine (5 min), followed by 2.5% (w/v) sodium thiosulphate solution (1 min) to remove residual colour. Sections were treated with 3% H_2_O_2_ for 15 min to block endogenous peroxidase activity. After deparaffinisation in xylene, sections were incubated in 0.3% H_2_O_2_ in methanol for 30 min. Non‐specific binding was blocked using normal goat serum (30 min, room temperature, humidity chamber). Sections were incubated at 37°C for 120 min with guinea pig–raised anti‐porcine TSHβ antibody (dilution 1:50), generously provided by Dr. A.F. Parlow (National Hormone and Peptide Program, NIDDK, USA). After washing with Tris‐buffered saline (TBS, pH 7.4) three times (5 min each), slides were incubated for 30 min with biotinylated goat anti‐guinea pig IgG (KPL, Cat. No. 71‐00‐30). Following another three TBS washes, sections were incubated with a streptavidin–phosphatase complex (KPL, Cat. No. 71‐00‐45) for 30 min. Immunoreactivity was visualised using Histomark Red solution (KPL, Cat. No. 55‐69‐00). Finally, sections were rinsed with wash buffer, and excess streptavidin–phosphatase was removed by additional rinsing (5 min). Following the washing steps, tissue sections were treated for 10 min with a working solution of chromogen and DAB substrate (dilution 1:50). The slides were subsequently rinsed, air‐dried, and coverslipped using DPX mounting medium. Primary antibodies were applied at a dilution of 1:300. To verify the specificity of the immunostaining, negative control slides were prepared by replacing the primary antibody with 10% normal goat serum. Additional controls were carried out by incubating sections with secondary antibodies raised in a species other than guinea pig, which resulted in no detectable immunoreactivity (Figure 1).

### 2.5 Quantitative Morphometric Assessment

Morphometric analysis was carried out using a compound microscope, considering only thyrotrophs with complete cross‐sections and clear, non‐reactive nuclei. Six samples per group were examined, with five slides prepared from each sample and ten microscopic fields analysed per slide. Measurements included thyrotroph count, cell diameter, cell area, cell volume, nucleus diameter, nucleus area, and nucleus volume. Cell and nuclear diameters (µm) were recorded at two perpendicular points (widest axis and 90° angle) using ImageJ software (ImageJ 1.44P, NIH, Bethesda, MD, USA) and AutoCAD 2004. From these diameters, areas (*A* = π*r* ^2^) and volumes (*V* = 4/3π*r* ^3^) were calculated, following the method of Justin et al. (1999).

### 2.6 Blood Collection

Blood samples (10 mL) were collected via direct jugular venipuncture to minimise variability due to sampling site. Samples were drawn into heparinised tubes, centrifuged at 2500 rpm for 10 min, and plasma was stored at –20°C until hormone assays were conducted.

### 2.7 Hormonal Assays

Plasma thyroid stimulating hormone (TSH; μIU/mL) was measured using a TSH ELISA kit (Catalog No. DSL‐10‐5300; Diagnostic Systems Laboratories, Webster, TX, USA). Plasma triiodothyronine (T_3_; ng/mL) was quantified using an Enzyme Immunoassay kit (Lot No. RN15685; Cat. No. BC‐1005; BioCheck, Burlingame, CA, USA). Total thyroxine (T_4_; µg/dL) was measured using a T_4_ Enzyme Immunoassay kit (Lot No. RN‐21055; Cat. No. BC‐1007; BioCheck, Burlingame, CA, USA). All procedures were performed according to the manufacturers’ instructions.

The sensitivity (minimum detectable concentration) of the assays was 0.05 μIU/mL for TSH, 0.2 ng/mL for T_3_, and 0.5 µg/dL for T_4_. The intra‐assay coefficients of variation (CVs) for three reference samples (low, medium, and high concentrations) were <6%, <5%, and <7% for TSH; <5%, <6%, and <8% for T_3_; and <6%, <5%, and <7% for T_4_. Inter‐assay CVs were consistently below 10% for all assays.

### 2.8 Statistical Analysis

Data were expressed as mean ± standard error of the mean (SEM). Differences among groups were analysed using one‐way analysis of variance (ANOVA), and when significant, means were compared using a post‐hoc multiple comparison test. Statistical significance was set at *p* < 0.05. Means sharing the same superscript letters were considered not significantly different.

## 3 Results

According to Table 1, the mean cell count of adenohypophyseal thyrotrophs did not differ significantly across age groups or sexes. However, cell diameter, area, and volume were significantly greater in the oldest age group compared to the younger groups (*p* < 0.05). Similarly, nuclear dimensions (diameter, area, and volume) showed significant enlargement with advancing age, particularly in camels aged 11 years and above (*p* < 0.05). Across sexes, males generally exhibited larger nuclear dimensions than females, with significant differences observed in nucleus diameter, area, and volume (*p* < 0.05). Overall, the data indicate that thyrotroph morphometry is influenced by both age and sex, with the most pronounced changes evident in older camels.

**TABLE 1:** Mean adenohypophyseal thyrotroph parameters of camel (*Camelus dromedarius*) at different age groups (immunocytochemistry).

	2–4 years			5–10 years			11‐onward years			Overall mean	
Groups	Male	Female	Overall mean	Male	Female	Overall mean	Male	Female	Overall mean	Male	Female
**Cell count** **(number)**	7.85 ± 0.32	7.60 ± 0.34	7.73 ± 0.23	7.55 ± 0.32	7.40 ± 0.30	7.48 ± 0.22	7.70 ± 0.54	6.50 ± 0.30	7.10 ± 0.32	7.70 ± 0.23	7.17 ± 0.19
**Cell diameter** **(µm)**	12.00 ± 0.60	10.53 ± 0.35	11.27 ± 0.36^B^	11.70 ± 0.34	11.37 ± 0.44	11.53 ± 0.38^B^	13.20 ± 0.60	13.1 ± 0.55	13.53 ± 0.40^A^	12.3 ± 0.36	11.67 ± 0.28
**Cell area** **(µm^2^)**	121.42 ± 10.14	89.99 ± 4.83	105.71 ± 6.15^B^	116.89 ± 8.72	105.84 ± 7.71	111.36 ± 5.82^B^	144.94 ± 12.65	141.72 ± 12.48	143.33 ± 8.81^A^	127.75 ± 6.21	112.5 ± 5.69
**Cell volume** **(µm^3^)**	1088.37 ± 117.42	671.39 ± 63.39	879.88 ± 7.15^B^	1015.92 ± 93.41	866.68 ± 89.42	941.30 ± 64.84^B^	1419.31 ± 181.97	1369.39 ± 188.83	1394.35 ± 130.04^A^	1174.31 ± 79.92	969.15 ± 78.42
**Nucleus diameter** **(µm)**	5.13 ± 0.26	4.57 ± 0.27	4.85 ± 0.19^AB^	5.23 ± 0.25	3.60 ± 0.20	4.42 ± 0.19^B^	5.57 ± 0.34	5.13 ± 0.31	5.35 ± 0.23^A^	5.31 ± 0.17^A^	4.43 ± 0.17^B^
**Nucleus area** **(µm^2^)**	22.26 ± 2.00	18.10 ± 1.79	20.18 ± 1.36^B^	22.93 ± 2.05	11.10 ± 1.22	17.04 ± 1.41^B^	27.02 ± 0.27	22.89 ± 2.60	24.95 ± 2.09^A^	24.08 ± 1.45^A^	17.36 ± 1.23^B^
**Nucleus volume** **(µm^3^)**	86.18 ± 10.42	64.20 ± 8.74	75.29 ± 6.89^B^	90.00 ± 11.34	31.32 ± 5.17	60.66 ± 7.26^B^	121.61 ± 21.33	93.83 ± 14.81	107.72 ± 13.00^A^	99.26 ± 8.83^A^	63.11 ± 6.50^B^

*Note*: Means sharing similar letters (A, B, C, D) are not significantly different at *p* < 0.05.

*Indicates a statistically significant difference.

**FIGURE 2:**
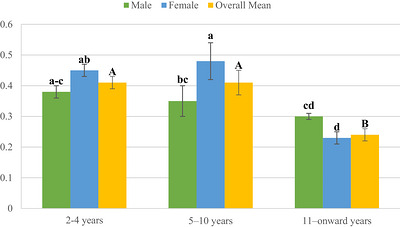
Mean plasma thyroid stimulating hormone in male and female camels (*Camelus dromedarius*).

As shown in Figure 2, plasma thyroid stimulating hormone (TSH) levels differed significantly among age groups (*p* < 0.05). The highest concentration was observed in females aged 5–10 years, followed by intermediate levels in the 2–4 year group. In contrast, the lowest TSH concentrations were recorded in camels aged 11 years and above. Overall, an age‐related decline in TSH was evident. Furthermore, females consistently exhibited higher values than males across all age groups.

**FIGURE 3:**
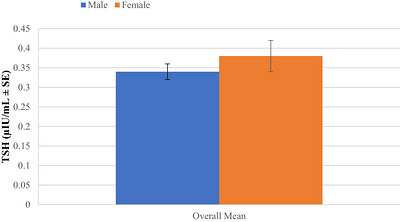
Overall mean plasma thyroid stimulating hormone in male and female camels (*Camelus dromedarius*).

Figure 3 shows the overall mean plasma TSH levels in male and female camels. Females exhibited higher mean TSH concentrations compared to males, although the difference was not statistically significant (*p* > 0.05).

**FIGURE 4:**
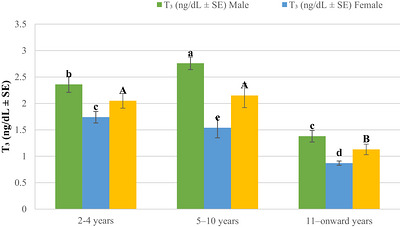
Mean plasma triiodothyronine in male and female camels (*Camelus dromedarius*).

Figure 4 shows that mean plasma triiodothyronine (T_3_) levels in camels varied significantly with age and sex. In the 2–4 years age group, males had higher T_3_ concentrations than females. A similar trend was observed in the 5–10 years group, where males showed significantly higher values than females. However, in the 11–onward years group, both sexes exhibited reduced T_3_ levels, with males showing the lowest values, while females maintained comparatively higher levels. Different superscripts indicate significant differences (*p* < 0.05) among age groups and between sexes.

**FIGURE 5:**
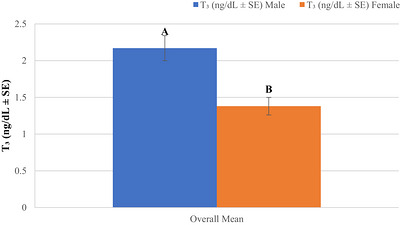
Overall mean plasma triiodothyronine in male and female camels (*Camelus dromedarius*).

Figure 5 shows the overall mean plasma triiodothyronine (T_3_) levels in male and female camels. Males had significantly higher T_3_ concentrations compared to females, as indicated by different superscripts (*p* < 0.05).

**FIGURE 6:**
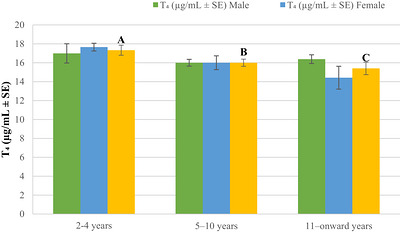
Mean plasma thyroxin in male and female camels (*Camelus dromedarius*).

Figure 6 shows that mean plasma thyroxin (T_4_) levels in camels were influenced by age but not markedly by sex. The highest T_4_ concentrations were recorded in the 2–4 years age group, followed by a decline in the 5–10 years group and the lowest levels in the 11–onward years group. Different superscripts (A, B, C) indicate significant differences (*p* < 0.05) among age groups, whereas values between males and females within each age group did not differ significantly.

**FIGURE 7:**
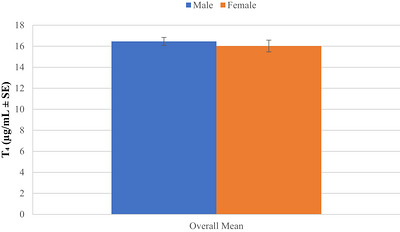
Overall mean plasma thyroxin in male and female camels (*Camelus dromedarius*).

Figure 7 illustrates the overall mean plasma thyroxin (T_4_) concentrations (µg/mL ± SE) in male and female camels. The mean T_4_ level in male camels was slightly higher than in females, with males showing a concentration of approximately 16.4 µg/mL and females around 16.0 µg/mL. However, the standard error bars indicate that this difference is minimal and may not be statistically significant. Thus, the overall mean thyroxin levels in male and female camels appear to be relatively comparable.

Figure 7 illustrates the overall mean plasma thyroxin (T_4_) concentrations in male and female camels. Although the mean T_4_ level in males appears slightly higher than in females, the difference was not statistically significant (*p* > 0.05).

## 4 Discussion

This study highlights how lactation, age, and sex influence thyrotroph morphology and thyroid hormone profiles in dromedary camels. The results demonstrate the adaptability of the hypothalamic–pituitary–thyroid (HPT) axis to physiological demands such as lactation and aging, while also revealing sex‐specific differences that add complexity to thyroid regulation in camels.

Lactation is one of the most metabolically demanding stages in mammals, requiring extensive endocrine adjustments to ensure adequate milk synthesis while maintaining maternal homeostasis (Khan et al. 2025). The present findings indicate that lactating camels exhibited a higher number of pituitary thyrotrophs, whereas non‐lactating camels demonstrated larger thyrotroph cell and nuclear dimensions. Morphologically, an increase in cell count may reflect proliferative adaptation or enhanced turnover of TSH‐producing cells, while enlarged nuclear and cytoplasmic areas in non‐lactating animals may suggest storage or accumulation of secretory products. Similar adaptations have been reported in other species, where lactation is associated with structural remodelling of anterior pituitary cell populations (Capuco et al. 2003; Lombardero et al. 2020).

The endocrine correlates of these morphological changes provide further insights. Plasma TSH concentrations were lower in lactating compared with non‐lactating camels, yet immunocytochemical analysis revealed an abundance of thyrotrophs in lactating animals. This apparent paradox may be explained by increased secretory turnover of TSH during lactation, resulting in smaller amounts of hormone retained within cells and lower circulating levels. In lactating rats, Ni et al. (2021) reported an upregulation of hypothalamic pro‐TSH mRNA during early lactation, followed by a decline, suggesting temporal modulation of TSH synthesis (Ni et al. 2021). These findings align with current observations, where lactation appears to be accompanied by an initial stimulatory drive of the HPT axis, followed by adjustments that prioritise efficient thyroid hormone utilisation rather than high systemic TSH levels.

Interestingly, lactating camels displayed higher circulating triiodothyronine (T_3_) levels despite reduced TSH and thyroxine (T_4_). This pattern strongly suggests an enhanced peripheral conversion of T_4_ to T_3_ via deiodinase activity, an adaptive mechanism widely described in mammals during lactation (Bianco and Kim 2006). The conversion of T_4_ into the metabolically active T_3_ ensures adequate energy metabolism to sustain milk production, even when TSH and T_4_ levels are relatively low. This lactation‐driven metabolic reprogramming may represent an evolutionary adaptation in camels, allowing them to maintain lactation under the energy constraints of desert environments.

Age‐related changes were also evident in thyrotroph morphology and thyroid hormone profiles. Younger camels (5–10 years) displayed higher T_3_ concentrations compared to older animals, consistent with the general decline in thyroidal activity with age documented in humans and livestock (Monzani et al. 1996 **;** Rahman et al. 2012). Morphologically, thyrotroph diameter, area, and nuclear dimensions were significantly greater in camels aged 11 years and above, suggesting a compensatory enlargement of cells despite lower functional output. This hypertrophy may reflect an attempt to sustain hormone production in the face of diminished secretory efficiency, a phenomenon also observed in aging pituitary tissues across mammalian species (Veldhuis 2013).

The interaction between lactation and age highlights an important feature of endocrine adaptation. While lactation in younger camels was clearly associated with elevated T_3_ and greater thyrotroph counts, this effect was less pronounced in older animals, where hormonal and morphological differences between lactating and non‐lactating individuals were attenuated. Such reduced plasticity of the HPT axis with advancing age may contribute to the diminished reproductive and lactational performance of older she‐camels, as has been reported in other domestic ruminants (Lucy 2001).

The inclusion of sex‐based comparisons further revealed distinct patterns in thyroid regulation. Male camels demonstrated significantly larger thyrotroph cell and nuclear profiles at 11 years and above, suggesting heightened pituitary activity compared with females. These morphological traits were associated with higher plasma T_3_ concentrations in males, particularly at younger ages. Elevated metabolic rates in males, especially during the breeding season, likely underlie these differences. The role of photoperiod and seasonal breeding patterns in camels may also influence thyrotroph activity, as seasonal modulation of pituitary function has been documented in other large herbivores.

Interestingly, while male camels exhibited significantly higher T_3_ concentrations and larger thyrotroph morphometry, no significant sex‐related differences were observed in plasma T_4_ concentrations (*p* > 0.05), as shown in Figure 7. This supports the idea that thyroxine serves as a relatively stable reservoir of thyroid hormone, with sex‐related differences more likely reflected in T_3_ production and conversion. These findings contrast with those of Tajik et al., who also reported no sex differences in T_3_ levels in dromedaries (Tajik et al. 2013). However, they are consistent with studies in other livestock species, such as Turkoman horses, sheep, and water buffaloes (Eshratkhah et al. 2010 **;** Nazifi et al. 2003; Tajik et al. 2011), where T_4_ levels remained relatively constant across sexes.

Taken together, these findings suggest that thyrotroph morphology and thyroid hormone profiles are subject to dynamic regulation by lactation, age, and sex. The reduced TSH and T_4_ but elevated T_3_ in lactating camels underscores the importance of peripheral deiodinase‐mediated conversion of T_4_ to T_3_ as a key compensatory mechanism during milk production. Furthermore, the increased thyrotroph counts in lactating females may reflect heightened demand for thyroidal stimulation, even if circulating TSH is reduced, highlighting the complex feedback loops governing the HPT axis.

The enlarged thyrotrophs and nuclei observed in older camels suggest an age‐related decline in pituitary efficiency, whereby cellular hypertrophy attempts to compensate for reduced hormone output (Jaspal et al. 2025). Similarly, the heightened T_3_ concentrations and increased thyrotroph dimensions in males likely reflect their greater metabolic activity and potential seasonal endocrine modulation. The lack of significant sex differences in T_4_ levels reinforces its role as a stable endocrine reservoir, with T_3_ production being more finely regulated to meet sex‐ and age‐specific metabolic demands.

The present study demonstrates that lactation in camels is associated with a functional reorganisation of the HPT axis, characterised by lower TSH and T_4_ but enhanced T_3_ availability. Age modifies these responses, with younger camels showing greater plasticity, while sex introduces additional variation, with males displaying heightened thyrotroph activity and T_3_ output. These findings provide valuable insights into the endocrine strategies employed by camels to sustain lactation and adapt to age‐ and sex‐related metabolic demands, and they align with the broader understanding of pituitary–thyroid interactions in mammals.

## 5 Conclusion

This study provides comprehensive insights into the morphological and hormonal dynamics of the hypothalamic–pituitary–thyroid (HPT) axis in dromedary camels, emphasising the influence of lactation, age, and sex. The findings demonstrate that lactation is associated with increased thyrotroph cell numbers and elevated plasma T_3_ concentrations, despite reduced TSH and T_4_ levels, suggesting enhanced peripheral conversion of T_4_ to T_3_ as a key adaptive mechanism. Age‐related changes revealed increased thyrotroph cell and nuclear dimensions in older camels, likely reflecting compensatory hypertrophy in response to declining endocrine function. Sex differences were evident in T_3_ levels and thyrotroph morphology, with males showing greater metabolic activity, while T_4_ levels remained stable across sexes. Overall, the study highlights the plasticity of the HPT axis in camels and underscores its critical role in maintaining metabolic and reproductive homeostasis under varying physiological demands.

## Author Contributions

**Shabana Naz**: writing – review and editing, writing – original draft. **Ibrahim A. Alhidary**: funding acquisition, resources. **Robina Shaukat**: investigation, validation, methodology. **Shaukat Ali Shaukat Jaspal**: conceptualisation, investigation, methodology, software, data curation. **Muhammad Mubashar Shaukat**: formal analysis, validation, visualisation. **Tahmina Shaukat**: formal analysis, software, data curation, resources. **Antonella Perillo**: writing – review and editing, writing – original draft. **Rifat Ullah Khan**: writing – review and editing, writing – original draft. **Naseer Khan Momand**: writing – review and editing, writing – original draft.

## Funding

This research was funded by the King Saud University, Riyadh, Saudi Arabia, through the Ongoing Research Funding Program (ORF‐2026‐833)

## Ethics Statement

The study was approved by the Ethical Committee of Faculty of Animal Husbandry & Veterinary Sciences, The University of Agriculture, Peshawar, Pakistan (Approval No. 12/FAH&VS/2021). Chatgpt has been used for English language

## Conflicts of Interest

No potential conflict of interest was reported by the authors.

## Data Availability

The relevant data are provided in the paper. The data of the current experiment can be obtained from corresponding author when needed.
